# Psychological factors in patients with peptic ulcerand functional dyspepsia

**Published:** 2014

**Authors:** Mahbobeh Faramarzi, Farzan Kheirkhah, Javad Shokri-Shirvani, Shokofeh Mosavi, Soroush Zarini

**Affiliations:** 1 Social Determinants of Health Research Center, Babol University of Medical Sciences, Babol, Iran; 2 Department of Psychiatry, Babol University of Medical Sciences, Ayatollah Rouhani Hospital, Babol, Iran; 3 Department of Gastroenterology, Babol University of Medical Sciences, Ayatollah Rouhani Hospital, Babol, Iran.; 4 Babol University of Medical Sciences, Babol, Iran

**Keywords:** Peptic ulcer disease, Functional dyspepsia, Conflict management, Psychiatric symptoms, Alexithymia.

## Abstract

***Background: ***The role of psychological factors in peptic ulcer disease (PUD) and functional dyspepsia (FD) has not been clearly determined. In this study the role of conflict management styles, psychiatric symptoms, and alexithymia were assessed in patients with PUD and FD and in the healthy individuals.

***Methods:*** Ninety subjects [30 PUD (15 women, 15 men), 30 FD (15 women, 15 men), and 30 healthy individuals (15 women, 15 men)] in two endoscopy wards of Babol University of Medical Sciences were evaluated. Three groups were matched with regard to demographic variables. Conflict management styles, psychiatric symptoms, and alexithymia were evaluated by appropriate questionnaires.

***Results:*** The patients with PUD reported less mean scores on psychiatric symptoms than the FD patients (depression 12.6±7.5 vs 28±9.5, anxiety 8.2±5.9 vs 18.7±6. obsessive-compulsive disorder 15.7±7.5 vs 21.8±8.4, interpersonal sensitivity 9.5±7.4 vs 16±7, psychoticism 8.03±4.5 vs 14.3±6.3, somatization 12.5±10.8 vs 20.7±8.1, and the total score of psychiatric symptoms 94.4±49.9 vs 160.1±46.6). The mean scores use of unconstructive conflict management styles in PUD patients were lower than FD (dominating 17.7±3.5 vs 20.2±2.7, avoiding 17.5±3 vs 23.8±4.4). Alexithymia symptoms were higher in FD patients than PUD individuals (difficulty in identifying feelings 23.5±6.3 vs 27.8±3.9, difficulty in describing feeling 16.5±4.4 vs 17.3±3.6). The PUD and FD patients had higher scores regarding these variables than the healthy subjects.

***Conclusion: ***The results show that both PUD and FD patients experienced more psychiatric symptoms, unconstructive conflict management styles, and alexithymia than the healthy subjects. FD patients had worse psychiatric problems than PUD.

Dyspepsia refers to pain or discomfort centered in the upper abdomen (1). It is a very common symptom worldwide that its investigation usually leads to two opposite diagnosis: peptic ulcer disease (PUD) or functional dyspepsia (FD). The prevalence of dyspepsia has been reported to range from 16-29% in Western countries to 8.5%-30% in Iran (2, 3). Dyspepsia is a common cause of health care utilization, resulting in increased medical costs as well as to individuals and society due to absenteeism and impairment of quality of life (4). Traditionally, PUD has been considered to be a psychosomatic disease. A recent evidence in 2013 supported that there is a relationship between mental disorder and the onset of self-reported peptic ulcer (5). Goodwin in 2009 found that mood/anxiety disorders are associated with increased rates of PUD.

Also, nicotine and alcohol dependence seem to play a substantial role in explaining the link with PUD (6). A recent systematic review has revealed that PUD signiﬁcantly impairs well-being and aspects of health-related quality of life, and is associated with high costs for employers and healthcare systems (7). Several psychological factors had indicated a link to PUD patients pointed out in previous literature, such as personality, stress, and addiction (8, 9). However, a review of literature on the subject reveals that information regarding psychological factors in Iranian patients with PUD is few. 

Functional dyspepsia (FD) defines as the presence of one or more dyspeptic symptoms (epigastric pain, early satiation, postprandual fullness, epigastric burning) considered to originate from the gastroduodenal region in the absence of any organic, systemic, or metabolic disease likely explains the symptoms (1). There is comorbidity between FD and mental disorders, especially mood and anxiety disorders (10). Alexithymia describes individuals who have difficulty identifying and describing their emotions in words and who instead focus on the details of external events (11). 

Although some research compared the psychological factors of PUD to FD (12), there is no published study to compare alexithymia and conflict management styles in two diseases. Also, little research is available regarding the role of psychological factors in Iranian population with FD or PUD diseases (13). The aim of this research was to compare the psychiatric problems of FD to PUD patients. We compared FD patients, PUD patients and the healthy individuals in three dimensions, conflict management styles (integrating, obliging, dominating, avoiding, and compromising), psychiatric symptoms (depression, anxiety, somatization, interpersonal sensitivity, obsessive-compulsive disorder, paranoid ideation, hostility, phobic anxiety, psychoticism), and alexithymia (difficulty in identifying feelings, difficulty in describing feelings, externally oriented thinking). 

## Methods

The adult patients with PUD and FD were recruited from the gastrointestinal endoscopy at two teaching hospitals of Babol University of Medical Sciences between April 2012 to March 2013. The subjects were screened by a medical student who associated the study to determine their recruitment eligibility. The patients with first experience of dyspeptic symptoms were entered into the study. Thus the patients with history of previous diagnosis of PUD and gastric surgery were excluded. All subjects were 20 to 65 years old and had earned more than primary education. Biochemical, ultrasonographic, and endoscopic examinations were performed to differentiate PUD/FD. At first, the patients were assessed for inclusion criteria (dyspeptic symptoms, age, and education) and clinical tests, they patients were referred to a gastroenterologist to confirm their PUD/FD diagnosis. The patients with gastroesoghageal reflux, biliary tract disease, gastric erosion, and gastric cancer were excluded. 

In patients without any structural gastrointestinal diseases, Rome III criteria were applied to identify FD (14). Thus, 30 patients (15 women, 15 men) diagnosed as FD were recruited. The gastrointestinal endoscopy allows physician to visualize ulcer in patients diagnosed as PUD (15). Thirty patients (15 women, 15 men) with PUD who were matched with FD groups in age, gender, education and marital status were entered into the study. The control group consisting of 30 persons (15 women, 15 men) with no history of psychiatric or gastroenterological disorder or current symptoms, and similar to the FD / PUD groups in age, gender, education and marital status, were selected from the patients admitted to other Outpatient clinics during the study period. Written informed consent was obtained from all subjects. All 90 subjects were asked to fill in the Rahim Organizational Conflict Inventory-II (ROCI-II), Symptom Checklist-Revised (SCL-90-R), and 20-item Toronto Alexithymia Scale (TAS-20). The protocol was approved by the Medical Ethics Committee of Babol University of Medical Sciences. 

**Questionnaires:**

**SCL-90-R** **:** The psychiatric symptoms were assessed with the Symptom Checklist-Revised (SCL-90-R). It is a self-rating inventory with 9 subscales for depression, anxiety, obsessive-compulsiveness, somatization, interpersonal sensitiveness, hostility, phobic anxiety, paranoid ideation, and psychoticism. The total scores are considered to measure the overall psychiatric symptoms. The persian version of the SCL-R was used in this study (16).

**ROCI-II: **The questionnaire is made up of 28 items, each item is rated on a 5 point likert scale anchored at 1=strongly disagree and 5=strongly agree. The higher the score, the greater the proportion of use of the conflict style. The five styles are labeled *integrating* (solving a problem together), *obliging* (accommodating the wishes of the others), *dominating* (using one’s influence to get one’s ideas accepted), *avoiding* (trying to keep disagreements with others to oneself in order to avoid hard feelings), and *compromising* (proposing a middle ground for breaking deadlocks). The subscales define two conflict management methods; constructive (integrating and obliging) and unconstructive (dominating and avoiding). The internal consistency index (α=0.50-0.81) and test-retest reliability (α=0.60-0.83 for the intraclass correlation coefficients) were acceptable (17). A valid Persian version of the ROCI-II was used in this study (18).

**TAS-20:** The TAS-20 is a self-report that is comprised of a 20-item Toronto Alexithymia Scale (TAS-20), which was widely used in the research. This questionnaire contains 20 items and 3 subscales that cover difficulty in identifying feelings (DIF, 7 items), difficulty in describing feelings (DDF, 5 items), and externally oriented thinking (EOT, 8 items). The subscale scores vary from 1 (strongly disagree) to 5 (strongly agree). Some items are reverse scored. The Persian version of TAS-20 was applied in this study (19).

Three groups were compared in age variable using ANOVA test and education status with χ^2^ test. All variables were entered in a multivariate analysis of variance model (MANOVA). A significant level was considered P<0.05.

## Results

The mean age of patients with PUD, FD and control group was 34.2±10.1, 33.03±10 and 36.8±8.8, respectively (P=03). Regarding the educational level, 18 (60%), 16 (53.3%) and 14 (46.7%) were undergraduate in PUD, FD and control group, respectively (0.6).

Table 1 shows the mean scores of the three subgroups on the ROCI-II, SCL-90, and TAS-20. The results of multivariate analysis of variance are shown in table 2. 

**Table 1:** Psychological profile of patients with PUD, FD, and healthy group

**Healthy** **Mean, SD**	**FD** **Mean, SD**	**PUD** **Mean, SD**	**Variables**
			**SCL-90**
6.8±5.3 4.9±4.2 6.1±5.8 6.6±4.2 5.7±3.9 6.2±3.8 5.4±3.2 3.1±3.4 2.2±2.8 50.3±30.1	28.0±9.5 18.7±6.03 20.7±8.1 21.8±8.4 16±7 14.3±6.3 10.2±3.6 9.9±4.8 6.5±4.7 160.1±46.6	12.6±7.5 8.2±5.9 12.5±10.8 15.7±7.5 9.5±7.4 8.03±4.5 9.8±4.2 7.5±5.2 3.3±2.7 94.4±49.9	Depression Anxiety Somatization Obsessive-compulsion Interpersonal sensitivity Psychoticism paranoid ideation Hostility phobic anxiety Total score
			**ROCI-II **
29.5±4 22.4±3.9 16.3±4.6 16.2±2.7 20.5±4.8	19.7±3.6 17.6±3 20.2±2.7 23.8±4.4 13.6±2.6	29±2.9 18.5±2.7 17.7±3.5 17.5 ±3 16.5±3.1	Integrating Obliging Dominating Avoiding Compromising
			**TAS-20**
20.6±4.3 14.6±3.6 20.4±4.3 55.6±10.4	27.8±3.9 17.3±3.6 17.8±3.7 62.9±4.7	23.5±6.3 16.5±4.4 21.8±3.3 62.2±10.6	Difficulty identifying feelings Difficulty describing feelings Externally-oriented thinking Total score

MANOVAs on SCL-90 subscales revealed a significant effect for the groups in all psychological symptoms (p<0.001). To compare with the controls, both PUD and FD patients had significantly higher mean of depression, somatization, obsessive-compulsion, hostility, paranoid, and the total score of SCL-90. Also, FD patients had significantly higher mean in all of the subscales of SCL-90 more than the control group. Significantly, higher scores were found in patients with FD compared to PUD for most psychiatric symptoms (depression, somatization, anxiety, obsessive-compulsion, interpersonal sensitivity, psychoticism, phobia, and the total score of psychiatric symptoms.

**Ranges of scores**: depression, 0-52; anxiety, 0-40; Somatization, 0-48; obsessive-compulsion, 0-40; interpersonal sensitivity, 0-36; psychoticism, 0-40; paranoid ideation, 0-24; hostility, 0-24; phobic anxiety, 0-28; total score of SCL-90; 0- 360. Integrating, 1-35 obliging, 1-30; dominatiing, 1-25; avoiding, 1-30; compromising 1-20. difficulty identifying feelings, 1-35; difficulty describing feelings, 1-25; externally-oriented thinking, 1- 40; total score of TAS-20, 1-100; MANOVAs on ROC-II subscales revealed a significant effect for the two groups in all conflict- management ways. Pairwise comparisons with Bonferroini test showed that PUD patients had lower mean of obliging and compromising score than the control group. FD patients had higher mean of dominating and avoiding more than the control group. Also, FD patients had lower mean of integrating, obliging, and compromising score than the control group. Lower scores were found in patients with PUD compared to FD for dominating, avoiding, and compromising score. Also, FD patients had lower mean of integrating score than PUD group. MANOVAs on TAS-20 subscales revealed a significant effect for the groups in all subscales and total scores. Pairwise comparisons revealed higher total alexithymia scores and DIF in PUD patients than healthy individuals. There was no difference between PUD and healthy groups in terms of DDF and EOT. Higher scores were found in patients with FD compared to healthy group for DIF, DDF, and total alexithymia scores. There was no difference between FD and healthy groups in term of EOT. Significantly, lower scores were found in patients with PUD compared to FD for DIF, DDF, and total alexithymia scores (p<0.05). 

**Table 2:** Results of Multivariate analysis variance of psychological factors between three groups

**Significant**	**F**	**Adjusted R ** ^2^	**Mean Square**	**Variables**
				**SCL-90**
<0.001 <0.001 <0.001 <0.001 <0.001 <0.001 <0.001 <0.001 <0.001 <0.001	62.342 52.656 22.385 36.220 20.537 21.974 16.154 17.274 11.461 49.356	0.580 0.537 0.320 0.442 0.305 0.320 0.254 0.268 0.190 0.521	3625.644 3094.867 3227.622 3492.467 1635.467 1079.489 434.467 707.489 285.356 183261.667	Depression Anxiety Somatization Obsessive-compulsion Interpersonal sensitivity Psychoticism paranoid ideation Hostility phobic anxiety Total score
				**ROCI-II **
<0.001 <0.001 <0.001 <0.001	72.195 19.173 8.364 41.910 27.456	0.615 0.290 0.142 0.479 0.373	1816.156 398.467 230.689 994.400 726.067	Integrating Obliging Dominating Avoiding Compromising
				**TAS-20**
<0.001 0.028 <0.001 0.004	16.069 3.742 8.470 5.961	0.253 0.58 0.144 0.100	786.422 114.689 244.467 965.622	Difficulty identifying feelings Difficulty describing feelings Externally-oriented thinking Total score

## Discussion

The results revealed that although psychiatric symptoms (depression, anxiety, obsessive-compulsion, interpersonal sensitivity, psychoticism, and hostility) were higher in both PUD and FD than in the controls, the mean of many psychiatric symptoms was higher in the FD than in the PUD. There is no published study to compare PUD and FD in psychiatric symptoms with SCL-90. Some studies reported psychiatric symptoms in FD patients. Faramarzi et al. in 2012 reported that significantly higher scores were found in patients with functional dyspepsia when compared with controls for most psychiatric symptoms; depression, anxiety, obsessive‑compulsive disorder, interpersonal sensitivity, psychoticism, hostility, and total score of psychiatric symptoms (20). A study reported significantly higher scores in FD patients compared to controls for all psychiatric symptoms, except psychoticism (21). Nakao et al. 2007 reported that state-trait anxiety score was signiﬁcantly higher in the FD group than in the peptic ulcer group (16).

According to our results, total alexithymia score of PUD was higher than controls, and similar to FD. Also, alexithymia scores of FD patients were higher than PUD. Although there is no published study comparing alexithymia in PUD and FD patients, little is known about alexithymia in PUD or FD patients. One study reported that alexithymia symptoms (DIF and DDF) were higher in FD patients than healthy subjects (25). In this study, the score of externally-oriented thinking of FD or PUD patients and healthy controls were the same. In contrast with our results, one study reported that externally-oriented thinking of FD patients was higher than those of controls (14). High range of alexithymia symptoms in PUD and FD patients suggest that alexithymia may play a role in the formation or aggregation of experiencing FD or PUD.

Our data support the conclusion that although both PUD and FD patients use less constructive conflict management styles than the healthy individuals, the FD patients use more unconstructive conflict management styles than PUD. No study has previously been published to assess or compare the conflict management styles in FD and PUD patients. One study reported that psychotherapy improved gastrointestinal symptoms and conflict management strategies in patients with functional dyspepsia (25).

Because of several limitations, generalization from the results should be made with caution. The study was a cross-sectional study, so causal relationship could not be determined. Also, some data gathered were related to patients with previous diagnosed PUD. A prospective study is proposed to confirm our results. Another weakness in our study is related to sampling. We recruited the patients from endoscopy ward of two teaching hospitals. Probably, the patients with moderate to severe PUD were entered into the study. Therefore, the results are not generalized to all FD/PUD patients, especially with mild illness. Further research is suggested with a multicenter and larger sample size. In conclusion, the present study shows that unconstructive management styles, psychiatric symptoms, and alexithymia are prominent and should be addressed in patients with PUD and FD.

## Notes

**Funding: **This paper is retrieved from a doctoral thesis. The Deputy Research of Babol University of Medical Sciences supported the funding (Grant Number 9031940).
